# Transcriptomic profiling of “brain-eating amoeba” *Naegleria fowleri* infection in mice: the host and the protozoa perspectives

**DOI:** 10.3389/fcimb.2024.1490280

**Published:** 2024-12-16

**Authors:** Vincent Guerlais, Nina Allouch, E. Ashley Moseman, Alicja W. Wojciechowska, Jakub W. Wojciechowski, Isabel Marcelino

**Affiliations:** ^1^ Institut Pasteur de la Guadeloupe, Les Abymes, Guadeloupe, France; ^2^ Department of Integrative Immunobiology, Duke University School of Medicine, Durham, NC, United States; ^3^ Department of Biomedical Engineering, Faculty of Fundamental Problems of Technology, Wroclaw University of Science and Technology, Wrocław, Poland; ^4^ Sano Centre for Computational Medicine, Cracow, Poland

**Keywords:** *Naegleria fowleri*, virulence phenotypes, mouse brain infection, RNA-seq analysis, neuroinflammation, host-parasite interaction, neurodegeneration, differentially expressed genes (DEGs)

## Abstract

The free-living amoeba *Naegleria fowleri* (NF) causes a rare but lethal parasitic meningoencephalitis (PAM) in humans. Currently, this disease lacks effective treatments and the specific molecular mechanisms that govern NF pathogenesis and host brain response remain unknown. To address some of these issues, we sought to explore naturally existing virulence diversity within environmental NF isolates. Herein, we purified two new NF environmental isolates (NF45 and NF1) and tested their *in vivo* virulence using experimental infection in mice. We found that NF45 was highly virulent (NF45_HV) compared with NF1 (low virulence, NF1_LV), based on *in vivo* amoeba growth kinetics and mouse survival. To identify underlying differences, we conducted RNA-seq and bioinformatics analyses from the infected mouse brains. Our results showed that NF1_LV and NF45_HV modulated the expression of their genes during mouse brain infection. Differentially expressed genes (DEGs) in NF1_LV were mostly involved in Translational protein, Protein-binding activity modulator, Protein modifying enzyme, while DEGs in NF45_HV were related to DNA metabolism, Cytoskeletal protein, Protein-binding activity modulator. Proteases (namely the virulence factor Cathepsin B) were upregulated in NF1_LV, while downregulated in NF45_HV. When analyzing the host response against infection by these two NF strains, enrichment analyses uncovered genes and mechanisms related to the host immune responses and nervous systems. We detected more DEGs in NF1_LV infected mice compared to NF45_HV, related to blood brain barrier leakage, immune cell recruitment, cytokine production (including IL-6, IFN-Ɣ and TNFα), inflammation of astrocytes and microglia, and oligodendrocyte and neurons degeneration. Increased expression of neuromotor-related genes such as *Adam22*, *Cacnb4* and *Zic1* (activated by NF1_LV infection) and *ChAt* (activated by NF45_LV infection) could explain PAM symptoms such as muscle weakness and seizures. Globally, our results showed that NF isolated from the environment can have different levels of virulence and differentially modulate their gene expression during brain infection. We also provided, for the first time, a comprehensive information for the molecular mechanisms of neuro-immune and host–pathogen interactions during PAM disease. As the host and the protozoa are strongly implicated in PAM lethality, new therapies targeting both the parasite, and the host should be considered to treat PAM infection.

## 1 Introduction

Protozoan parasites represent a significant threat to health causing severe diseases in humans worldwide (Daumerie et al., 2010; Baldursson and Karanis, 2011; Andrews et al., 2014; Ma et al., 2022). Besides the widely known diseases caused by *Toxoplasma*, *Plasmodium* and *Leishmania*, other protozoa can cause fatal human diseases such as *Naegleria fowleri. N. fowleri* (NF), commonly called “brain-eating amoeba”, is a free-living amoeba causing one of the most devastating forms of meningoencephalitis known as primary amoebic meningoencephalitis (PAM). In the United States, *N. fowleri* is classified as a category B priority pathogen, the second highest class of priority biological agents (NIH: National Institute of Allergy and Infectious Diseases, 2023). It is also considered as an emerging neglected protozoa and a primary agent of infectious water-borne outbreaks (Daumerie et al., 2010). Approximately 500 cases of PAM have been reported worldwide, with most reported in the US (120 between 1978–2018), with only 7 survivors (Gharpure et al., 2021). Several cases have been recently reported in Pakistan (Nadeem et al., 2023), South Korea (Hong et al., 2023) and Israel (2024). This is likely an underestimation of the worldwide occurrence of PAM, as it is often mistaken for other common neuroinfections (Matanock et al., 2018). The amoeba resides in soil, warm fresh waters, hot springs, and waterparks and it is difficult to predict the presence and/or outbreaks in natural and treated waters (Moussa et al., 2013, 2015; Reynaud et al., 2020; Chaúque et al., 2022; Leal dos Santos et al., 2022; Vingataramin et al., 2024). PAM cases are expected to increase worldwide due to rising temperatures and extreme weather events (such as floods), reduced levels of chlorine in potable water, or deteriorating water distribution systems (Marciano-Cabral et al., 2003; Shakoor et al., 2011; Miller et al., 2015; Cooper et al., 2019; Cope et al., 2019; Maciver et al., 2020; Leal dos Santos et al., 2022; Nadeem et al., 2023; Ward and Sherchan, 2023; Vingataramin et al., 2024). Even though the infection usually originates through the practice of recreational aquatic activities, it can also occur through ablution practices performed by religious groups and hygiene devices like neti-pots (Siddiqui et al., 2016; Yousuf et al., 2024).

Infection occurs upon accidental introduction of *N. fowleri* trophozoite (the replicative and infectious form of the amoeba) into the nose, often after exposure to freshwater. The amoeba crosses the cribriform plate to reach the human brain and causes severe destruction of the central nervous system (CNS) resulting in cerebral oedema, necrosis, herniation, and, in most cases, death (De Jonckheere, 2002; Moseman, 2020). Early symptoms may include headache, fever, nausea, or vomiting and later symptoms can include stiff neck, confusion, lack of attention to people and surroundings, loss of balance, seizures, and hallucinations. After the start of symptoms, the disease progresses rapidly and usually causes death within about 5 days. This life-threatening infection is managed with a heavy drug regimen (Cope, 2013; Burki et al., 2024) but the mortality rate remains up to 97%. Differences in the degree of virulence, drug susceptibility, replication rate, gene content and expression levels have been reported amongst *N. fowleri* isolates (Cursons and Brown, 1978; John and John, 1989, 1994; Herman et al., 2021; Russell and Kyle, 2022; Dereeper et al., 2023) but there is limited knowledge on the mechanisms underlying NF pathogenesis. Plus, their impact on the mammalian host brain and on the host immune response is largely unknown.

Several studies have identified potential *N. fowleri* virulence-associated factors, and two primary mechanisms that contribute to the trophozoites invading the host CNS: contact-independent (brain damage through the release of proteases, for instance) and contact-dependent (brain damage through surface structures named food cups) (Jamerson et al., 2012; Herman et al., 2021; Rodriguez-Anaya et al., 2021; Sarink et al., 2022; Martínez-Castillo et al., 2024). Several NF gene expression profiling studies have been performed to understand the biology of *in vitro* derived low-versus high-pathogenicity NF (Zysset-Burri et al., 2014; Herman et al., 2021). However, the majority of studies have utilized clinical NF strains isolated in the 70’s and 80’s to understand *N. fowleri* virulence and infection process. These strains are cultured axenically *in vitro* (Joseph et al., 2021; Dereeper et al., 2023) which are known to decrease NF virulence (Wong et al., 1977; John and Howard, 1993) causing possible biases in genes expression with a likely biological impact via transcriptome changes (as observed in other protozoa (Bussotti et al., 2021; Santi and Murta, 2022; Black et al., 2023)).

Herein, we used recently isolated, low passaged NF strains to identify NF-virulence genes and study NF-host interaction. Since PAM is a rare disease, recent clinical samples are difficult to obtain, so we isolated environmental NF samples from recreational baths in Guadeloupe (Moussa et al., 2013; Vingataramin et al., 2024). After NF isolation from waters, we maintained them at a low passage number in axenic culture conditions and used them to infect mice. After *in vivo* NF-virulence phenotyping validation, we used high-throughput RNA sequencing and perform bioinformatics analysis of differential gene expression to compare the gene expression profiling of (i) the high and low virulent NF before and during the mouse brain infection and (ii) non-infected versus infected host brains with NF1_LV and NF45_HV to perform the first system-wide dual analysis of host and parasite gene expression. Further gene ontology, pathway and protein-protein network analyses provided insights into the higher-level processes activated during the infection process in both the host and the protozoa.

## 2 Materials and methods

### 2.1 *Naegleria fowleri* strains isolation, identification and culture

The two *Naegleria fowleri* strains used in this study were isolated in 2020 from environmental water samples collected in 2 different geothermal baths in Guadeloupe: *N. fowleri*_1 (NF1, isolated in Bain de Morphy, Bouillante) and *N. fowleri*_45 (NF45, isolated in Bain de Grosse Corde, Capesterre Belle Eau) (Vingataramin et al., 2024). Briefly, 1L water samples were filtered, and filters were inoculated onto non-nutritive agar with *Escherichia coli* ATCC 25922 (NNA-*E. coli*) at 40°C, as described elsewhere (Moussa et al., 2013). NF strains were maintained in culture on NNA-*E. coli* until monocultures of NF were observed. Amoeba identification was performed by PCR using ITS and 18S amplicons Sanger sequencing (Moussa et al., 2013; Reynaud et al., 2020; Vingataramin et al., 2024) at Eurofins Genomics (Germany). Genotyping of the strains, using the set of ITS defined (De Jonckheere, 2011) revealed a genotype 2 for both strains (**Supplementary Table S1**). Before animal experiments, NF1 and NF45 were cultured during only 5 passages in axenic cultures conditions using SCGYEM culture medium (De Jonckheere, 1977). The virulence of each strain was then established using a C57BL/6 mouse model, as described below.

### 2.2 Animal infections

*N. fowleri* strains were grown at 33°C, 5% CO_2_ in Nelson’s complete media (NCM) before mouse passage. *N. fowleri* were maintained in culture containing feeder cells with cell splitting and media replacement every 2-3 days. C57BL/6 mice (female and male) aged between 8 to 10 weeks were used for animal infection experiments. Mice were anesthetized using isoflurane prior to *N. fowleri* inoculation. Under anesthesia, mice received a total of 20μl of PBS containing 5x10^4^ *N. fowleri* trophozoites. 10μl of PBS inoculum was instilled into the nasal cavity by administering droplets to each naris and allowing the sedated animal to inhale the liquid droplet - no force was applied. After ensuring that the drop is inhaled, the mouse was put back in the cage and monitored until it wakened up. Animals were monitored for health status until reaching humane endpoints or being sacrificed at different timepoints post-infection for amoeba quantification, harvest, or histologic analysis. For histologic analysis, infected mice were intracardially perfused with 2% buffered Formalin, heads were decalcified, and 30 µm frozen sections made. Sections were stained with anti-amoeba antibody (2B6), CD45 (immune cells), DAPI (nuclei) and imaged on a Leica SP8 confocal microscope. Male and female mice were equally represented across experiments (no phenotypic differences between male and female mice were observed). All mice in this study were handled in accordance with the guidelines set forth by the Duke Animal Care and Use Committee.

### 2.3 Fluorescence activated amoeba quantification and sorting

Mice were anesthetized prior to intracardiac perfusion with saline solution to remove blood contamination after which brain regions were removed from within the skull. Tissue was minced with scissors, then digested in 0.5 mL Hibernate A with DNase I (Roche, 0.5 mg/mL) and Collagenase D (Roche, 0.5 mg/mL) at 37°C for 30 min with constant shaking. Cells were washed, spun down at 500 x g for 5 min, and resuspended in PBS. Cell suspensions were stained at 4°C for 20 min with antibodies amoeba cell surface antigen (2B6), then washed and spun down at 500 x g for 5 min. For quantification prior to flow cytometry analysis on BD Fortessa cells were fixed with 2% formalin and Precision Count beads (Biolegend) were added to each sample. For NF sorting, brain samples from mice infected with NF1_LV and NF45_HV were stained with 2B6 and CD45 and sorted on a Sony MA900 for 2B6+ CD45- cells. NF1_LV (n=3) and NF45_HV (n=3) inocula were used as a control group for NF before infection. Infected brain samples were collected at Days 3 and 5 for NF45 (total of 6 biological replicates) and at Day 5 for NF1 (3 biological replicates). Non-infected brain samples were collected and used as controls (n=3). All samples were frozen at –80°C until further RNA extraction.

### 2.4 Total RNA extraction and preparation

**Figure 1:**
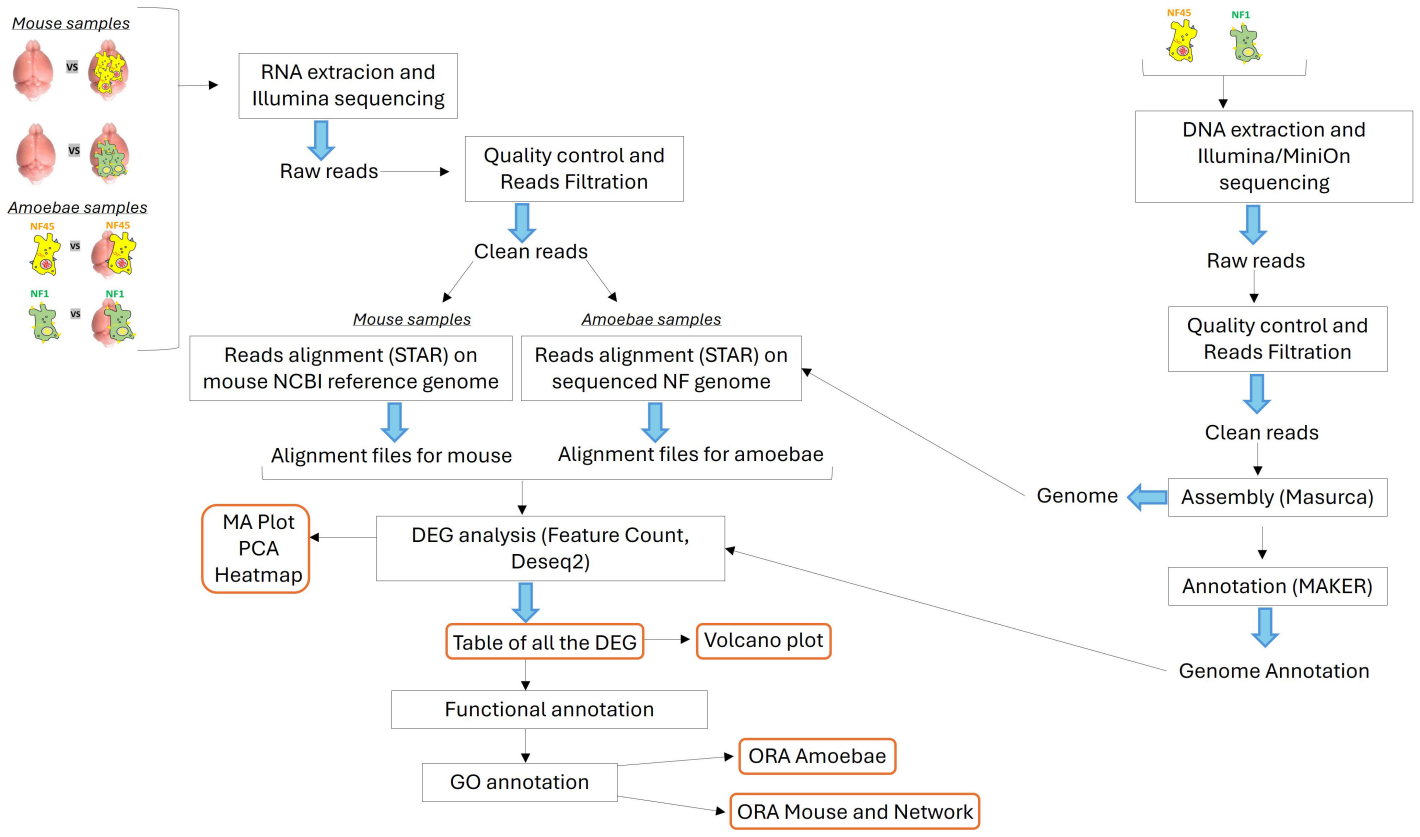
Schematic overview of the bio-informatic workflow conducted in this work. The central part depicts the transcriptomic workflow for both the NF samples and the mouse samples. The right part shows the genomic workflow for the genome assembly and annotation of NF1_LV and NF45_HV.

The RNA extraction, library preparations and Illumina sequencing were conducted at GENEWIZ (New Jersey, United States). The biological samples used for RNAseq analysis were as follows: (i) NF1_LV before and after brain infection, (ii) NF45_HV before and after brain infection, (iii) mouse brain infected and non-infected by NF1_LV and (iv) mouse brain infected and non-infected by NF45 (**Figure 1**).

### 2.5 *Naegleria fowleri* genome sequencing and annotation

DNA was extracted from both NF1_LV and NF45_HV strains (trophozoites), as previously described (Dereeper et al., 2023). The extracted DNA was subjected to sequencing using both Illumina and Oxford Nanopore (MinION) platforms to obtain short and long reads, respectively. The quality of the reads was assessed using FastQC (Andrews, 2010). Trimming and filtering of the reads were conducted with Cutadapt (Martin, 2011) and Trimmomatic (Bolger et al., 2014) to ensure high-quality reads for downstream analysis. The filtered reads were assembled *de novo* using the MaSuRCA assembler (Zimin et al., 2013), which combines short and long reads to produce a comprehensive genome assembly. Finally, the assembled genomes were annotated using the MAKER annotation pipeline (Holt and Yandell, 2011; Dereeper et al., 2023) (**Figure 1**).

### 2.6 Bioinformatics analysis of RNAseq

After removal of sequencing adapters with Cutadapt (Martin, 2011), the raw reads were filtered and trimmed using Trimmomatic (Bolger et al., 2014). These reads were then mapped to a database of unwanted RNA to remove rRNA or contaminant RNA (contaminating RNA from mouse in brain-isolated amoeba samples) with Bowtie2 (Langmead and Salzberg, 2012). The rRNA collection file was built using the Silva reference database (Quast et al., 2013). The final quality of reads was processed with FastQC (Andrews, 2010) and MultiQC (Ewels et al., 2016) to combine the resulting quality reports (**Figure 1**).

For NF1_LV and NF45_HV samples, the reads were aligned using STAR (Dobin et al., 2013) to the final previously assembled NF1 and NF45 genomes (see above) to generate alignment files. For mouse brain samples, the clean reads were aligned to the NCBI *Mus musculus* reference genome, GRCm39, Genome Reference Consortium, NCBI accession GCF_000001635.27 with STAR (Dobin et al., 2013) to generate alignment files. After NF and mouse alignment processes, the generated SAM files were converted to BAM files through SAMtools (v1.11). FeatureCounts (v2.0.1) was used to estimate read counts (Liao et al., 2014) for each identified genes. The resulting count tables were loaded in R (R Core Team, 2023) to use DESeq2 R package to perform differential gene analysis. (v1.42.0) (Love et al., 2014). The quality control of the RNAseq data was checked with MA-plots to analyze normalization bias, and principal component analysis (PCA) was performed to observe the clustering of each biological replicate. A heatmap plot was also generated to overview the gene expression profiles under the different conditions. Differential expression representation of genes was performed using Volcano plots (**Figure 1**).

### 2.7 Functional enrichment analysis

The functions of the differentially expressed genes (DEGs) were then assigned according to the best alignment of predicted protein sequences using BLASTP (default values: E-value = 1e− 03) to the Uniprot database (including the SWISS-PROT and TrEMBL databases). Each DEGs had a unique Uniprot identifier (**Figure 1**).

#### 2.7.1 *Naegleria fowleri* DEGs

Gene Ontology (GO) categories in NF1_LV and NF45_HV DEGs lists were identified using the PANTHER (Protein ANalysis THrough Evolutionary Relationships) (version 5.1.13) Classification System (https://www.pantherdb.org/about.jsp) (Mi et al., 2021), by mapping the DEGs to the gene ontology (GO) database. The Gene Ontology information for each DEG was extracted using homology transfer. GO terms which appeared in at least 10 DEGs in each strain was then further considered for functional enrichment analysis. For each GO term, a Fisher test was performed to examine if there is a significant difference in the number of genes associated between both strains. The statistical analysis was done with the scipy package available in Python 3.11. (Virtanen et al., 2020) and fisher_exact function with default parameters was used. The visualization of the results was performed with the Python matplotlib package (Hunter, 2007).

#### 2.7.2 Mouse brain DEGs

GO analysis was performed using the genome-wide annotation of mice (Carlson, 2019). The overrepresentation analysis (ORA) for GO Biological Process (BP), Molecular Function (MF) and Cellular Component (CC) was performed with the clusterProfiler package version 4.10 (Wu et al., 2021) available in R. The default parameters of the compareCluster (fun=enrichGO) function in clusterProfiler were used for identification of significant terms [p-value<0.05, applied Benjamini-Hochberg correction (Benjamini and Hochberg, 1995)]. The equivalent procedure was applied with respect to the Kyoto Encyclopedia of Genes and Genomes (KEGG) (Kanehisa et al., 2019) using compareCluster (fun=enrichKEGG) function with the same default parameters. Visualization of the results from ORA was supported by R package enrichplot version 1.22 (Yu, 2023) and ggplot2 (Wickham, 2011).

### 2.8 Protein-protein interaction network construction and detection of key genes involved in PAM infection

To further detect important candidate genes involved in NF infection, a network analysis was performed. Both mice DEGs datasets (either infected with NF1_LV or NF45_HV strains) were subjected to Cytoscape STRINGApp (Doncheva et al., 2019), with default parameters (confidence score > 0.4), to extract their protein-protein interaction (PPI) networks. The resulting networks were analyzed with respect to their degree distribution using the network package (Hagberg and Conway, 2020) version available in Python 3.11.

### 2.9 Data availability

The data presented in the study were deposited in the NCBI database under BioProject accession number PRJNA1181852. The raw RNA-seq reads and genome data can be accessed in the Sequence Read Archive (SRA) associated with this BioProject. Naegleria fowleri ITS and 18S sequences for NF1 and NF45 strains have also been deposited on NCBI and are available in GenBank under accession numbers: PQ573549, PQ573550, PQ571242 and PQ571243.

## 3 Results

### 3.1 NF1 and NF45 environmental strains display distinct natural virulence traits in C57BL/6 mice

**Figure 2:**
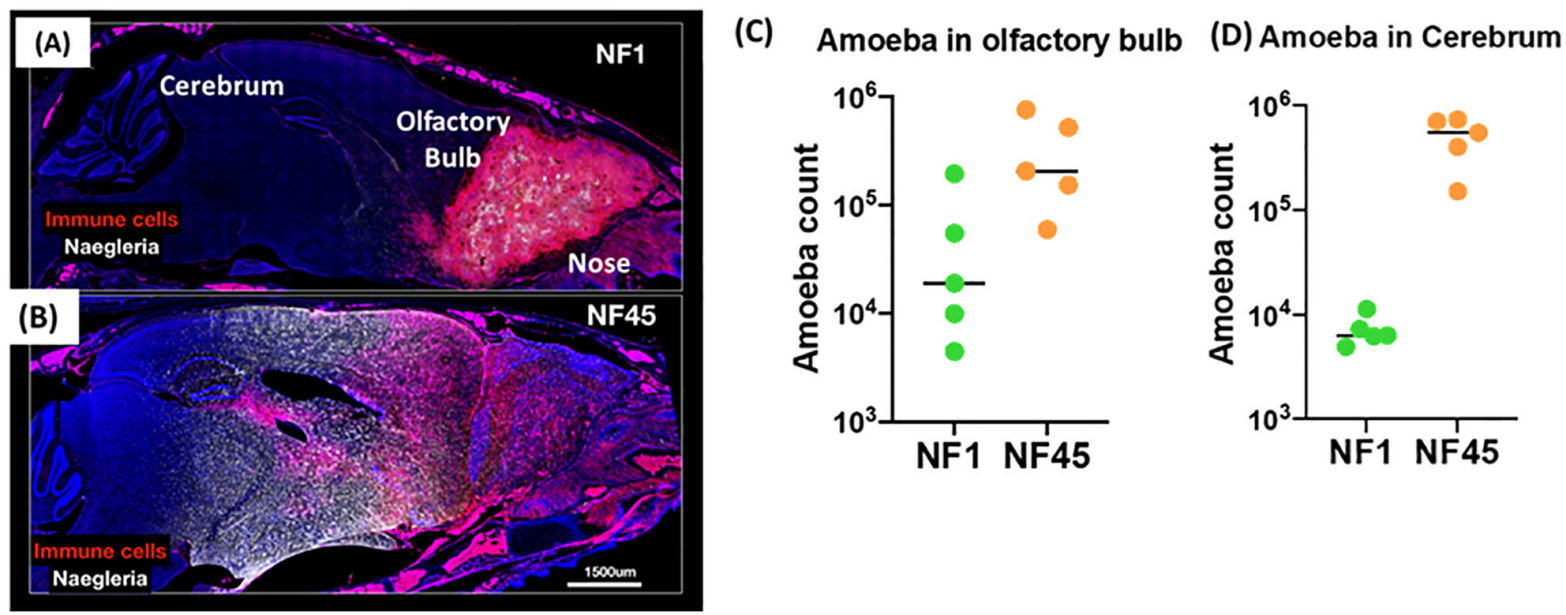
Virulence of NF1 and NF45 in the C57BL/6 brain at Day 4 after infection. Representative micrographs of the infected brains for experimental group infected by NF1 **(A)** or NF45 **(B)**. Parasitic loads in olfactory bulb **(C)** and cerebrum for **(D)** NF1 and NF45. NF1 is the low virulent strain (NF1_LV) while NF45 is the highly virulent strain (NF45_HV).

To evaluate the potential for the environmental isolates NF1 and NF45 to cause disease, we utilized a mouse model of infection. Mouse infection with NF1 and NF45 revealed marked differences in symptom onset and disease severity that indicate these two newly isolated *N. fowleri* strains display distinct virulence phenotypes. *In vivo* experiments showed that NF1 displayed a slower progression of the infection from the olfactory into deeper brain regions, with fewer parasites invading the cerebrum at day 4 post infection (**Figures 2A–D**). This was associated with observed average time to death at day 7 post-infection (data not shown). On the contrary, NF45 strain exhibited a rapid progression into the brain (**Figure 2B**), marked with high parasite load in the cerebrum (up to 100 x higher compared to NF1) (**Figure 2C**) at 4 days post-infection; the average time to death was of 5 days (data not shown). These results supported the use of the less virulent *N. fowleri* NF1 (NF1_LV) strain and the highly virulent *N. fowleri* NF45 (NF45_HV) strains for comparative transcriptomic analysis.

### 3.2 NF1_LV and NF45_HV strains exhibit different transcriptome profiles during brain infection

To explore *Naegleria fowleri* differential gene expression during mouse brain infection, we (i) compared NF1_LV and NF45_HV DEGs during and before infection and (ii) evaluated the transcriptome profiles of both amoebae strains. To accomplish this, NF1_LV and NF45_HV amoebae were sorted from infected mice at different timepoints post infection.

#### 3.2.1 Transcriptome profiling of DEGs in NF1_LV and NF45_HV strains

First, we performed MA-plots to observe the distribution of the genes according to fold-change and counts (**Supplementary Figure S1**). The results showed that there is no normalization bias. Then, we assessed the clustering between biological replicates using Principal Component Analysis (PCA) (**Supplementary Figures S2A, B**). For NF1_LV samples (**Supplementary Figure S2A**), PC1 and PC2 explained 83% and 12% of gene expression variation, respectively, and the samples were clustered in 2 different groups and could be used for DEGs identification. For NF45_HV samples (**Supplementary Figure S2B**), PC1 and PC2 explained 77% and 8% of gene expression variation, respectively, and the samples collected at D0, D3 and D5 were clustered in 2 major different groups (D0 and D3+D5) and could be used for DEGs identification. Heatmap illustrated that the gene expression patterns were similar within groups, while different between groups (**Supplementary Figure S2C**). Dark blue indicates a high gene expression similarity, while light blue indicates more differences in gene expression between these samples.

**Figure 3:**
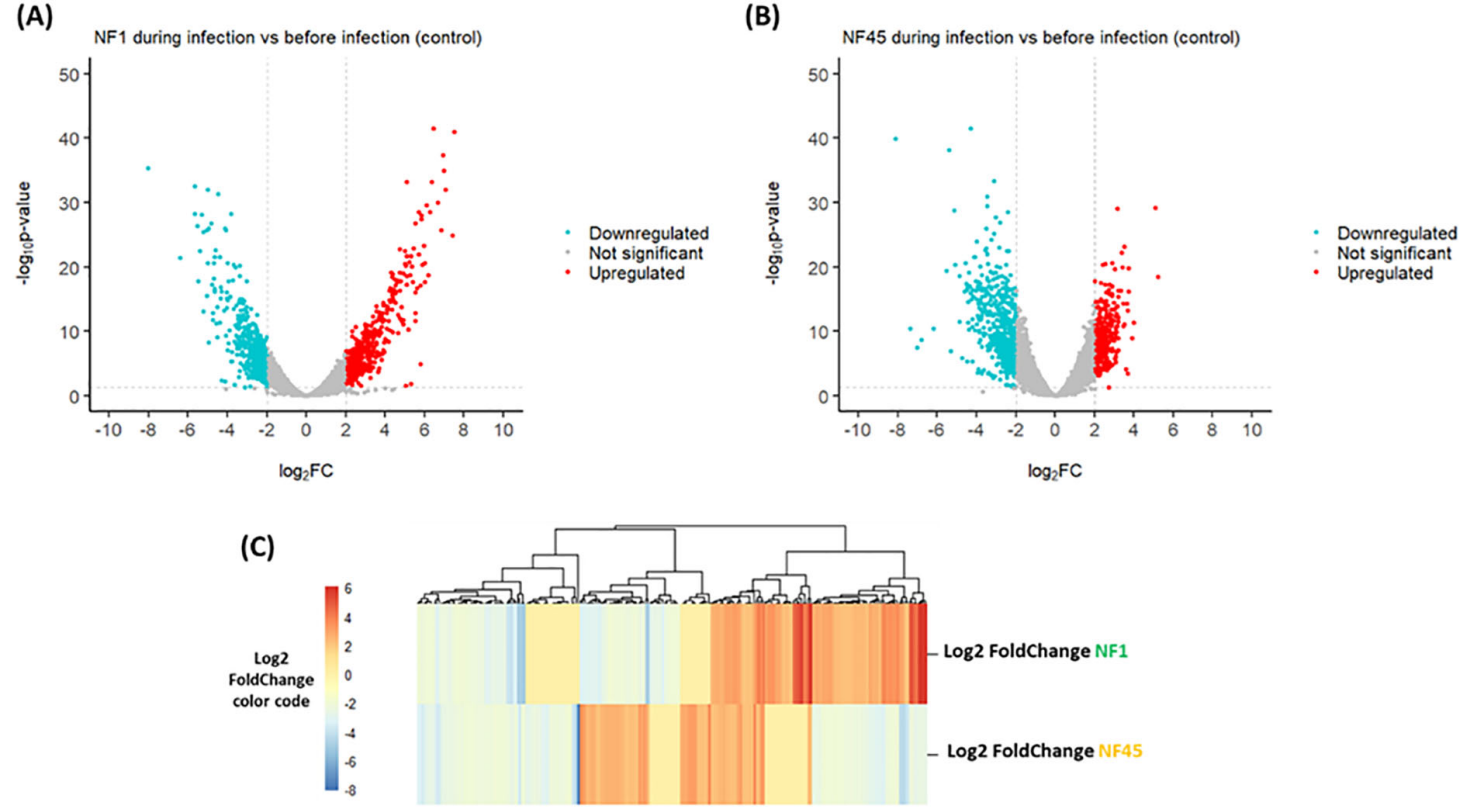
Differential Gene Expression analysis for both NF1_LV (NF1) and NF45_HV (NF45) in infected brains. Volcano plots of the up- and downregulated genes and unchanged genes for NF1_LV **(A)** and NF45_HV **(B)**. The up- and down-regulated genes are shown in color and the unchanged genes in grey. The dashed line indicates the threshold line for differential gene screening criteria. **(C)** Heatmap of common DEGs between NF1_LV and NF45_HV.

According to the criteria with p-value <0.05 and |log2FoldChange| ≥ 2, a total of 592 genes were differentially expressed during the infection in NF1_LV strain with 301 and 291 genes exhibiting up- and downregulated expression, respectively (**Supplementary Table S2**; **Figure 3A**). In NF45_HV, 585 genes were differentially expressed during the infection, with 252 and 333 genes exhibiting upregulated and downregulated expression, respectively (**Supplementary Table S2**; **Figure 3B**). The values revealed that only 5% of the total gene in *Naegleria* pangenome were modulated when NF reached the mammalian host brain. We observed that NF1_LV and NF45_HV shared 235 DEGs (approx. 40% of their DEGs) and that, in many cases among those common genes, up-regulated genes in NF1_LV were down–regulated in NF45_HV and vice-versa (**Figure 3C**; **Supplementary Table S2**).

#### 3.2.2 Functional enrichment of NF DEGs

**Figure 4:**
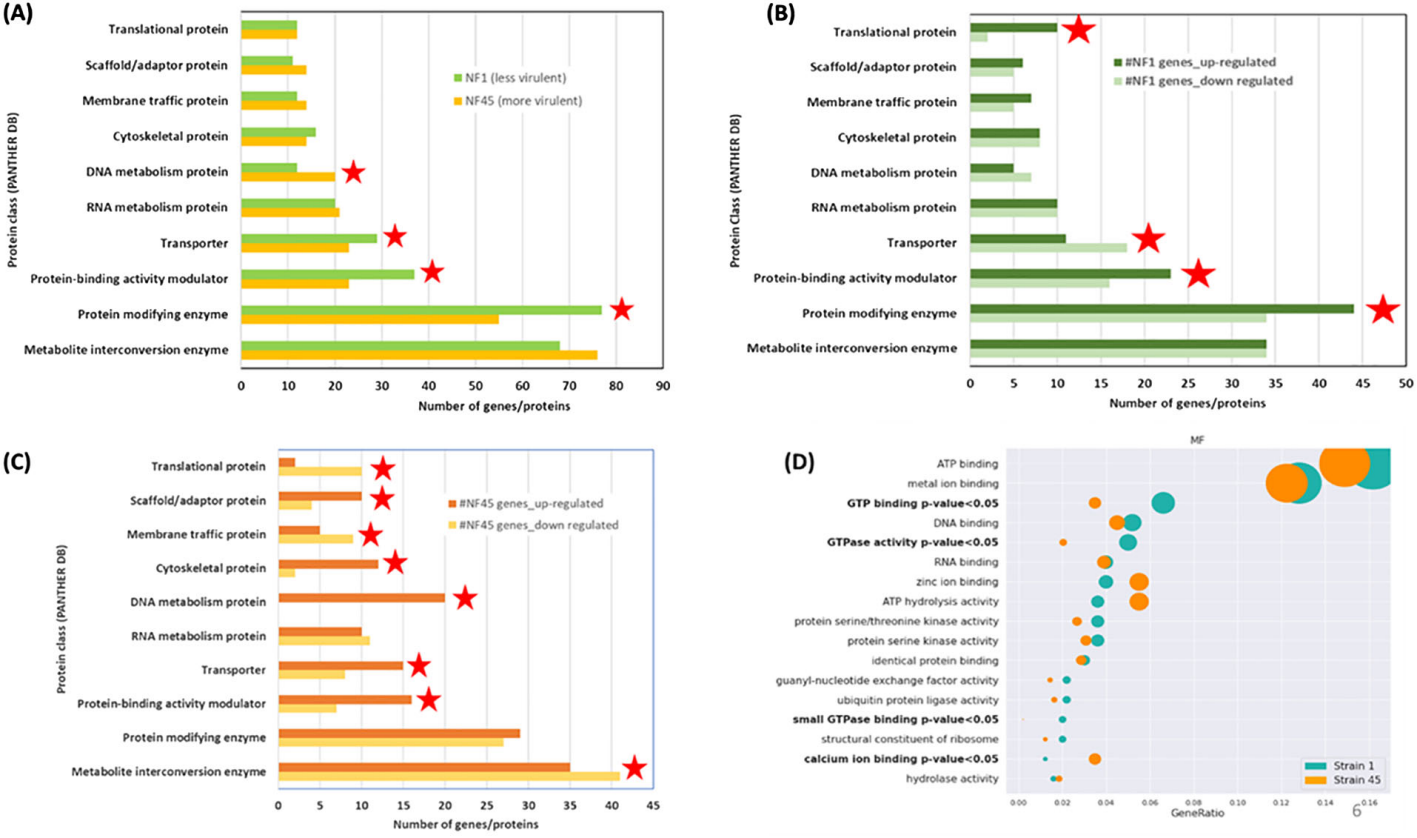
Functional enrichment analysis of differentially expressed genes (DEGs) in NF1_LV (green) and NF45_HV strains (orange). **(A–C)** show the PANTHER DB protein classes enrichment and **(D)** visualization of Fisher test results for GO terms, in bold terms with statistically significant difference in the number of proteins, the dot size relates to the number of proteins associated with the GO term. Red stars indicate the protein class with more DEGs.

To elucidate the biological function of the DEGs (**Supplementary Table S2**), we performed a Gene ontology (GO) using PANTHER database on the set of 577 DEGs using their unique Uniprot identifiers for NF1_LV and 567 for NF45_HV. As many *Naegleria* genes have unknown function (Dereeper et al., 2023), only 40% of the genes were included in the analysis. From this, we observed that the major differences in DEGs between NF45_HV and NF1_LV reside in 4 protein classes: DNA metabolism protein, Transporter, Protein-binding activity modulator and Protein modifying enzyme (**Figure 4A**).

**Figure 4B** revealed that when NF1_LV replicates in the brain, there was a strong modulation in the expression of genes involved in 4 classes: Translational protein, Protein-binding activity modulator, Protein modifying enzyme (all up-regulated during infection) and Transporter (down-regulated during infection). The top 5 most upregulated genes in NF1_LV included SF-assemblin (major component of the striated microtubule-associated fibers (SMAFs) in the flagellar basal apparatus), Carboxypeptidase A4 (involved in proteolysis), Intraflagellar transport protein 20 homolog (important for intracellular transport), Mitochondrial inner membrane protease atp23 (serves as a processing peptidase) and Histidine ammonia-lyase (Histidase) (**Supplementary Table S2**). Of the top 5 genes that exhibited downregulated expression in NF1_LV, Signal recognition particle receptor subunit beta (transmembrane GTPase), Diphthine methyltransferase, Serine/threonine-protein kinase Nek7 (which plays an important role in mitotic cell cycle progression), probable RNA-binding protein 19 (a nucleolar protein conserved in eukaryotes) and Replication factor A protein 1 (major single-stranded DNA binding factor) were detected.

Compared with *in vitro* growth, during NF45_HV brain replication we observed a difference in the expression of genes involved in 8 protein classes: Translational protein, Membrane-traffic protein, Metabolite interconversion enzymes (all down-regulated), Scaffold/adaptor protein, Cytoskeletal protein, Calcium-binding protein, DNA metabolism protein, Transporter, Protein-binding activity modulator (all up-regulated) (**Figure 4C**). Among the DEGs in NF45_HV, we highlighted the top 5 most upregulated genes Putative ariadne-like RING finger protein R811 (transferase), O-methyltransferase MdmC (antibiotic biosynthetic process), F-box/LRR-repeat protein 4 (autophagy of mitochondrion), proteasome subunit alpha type-5 (involved in the proteolytic degradation of most intracellular proteins) and Conditioned medium factor (CMF) (Density-sensing factor). The top 5 most downregulated genes include Cathepsin B-like CP3 (protease considered as a virulence factor in NF), Dynein axonemal heavy chain 8, Leucine-zipper transcription factor-like protein 1 (both involved in cilia motility), WD repeat-containing protein 54 (the three genes being involved in cilia motility) and protein angel homolog 2 (involved in the regulation of mitotic cell cycle).

Additionally, we performed a GO over-representation (ORA) analysis (using the annotation obtained from PANTHER DB) to infer a set of modulated biological pathways or processes in which certain DEGs either in NF1_LV or NF45_HV could play a significant role during the infection process. For this, we selected only the significant GO terms appearing at least in 10 genes in either NF1_LV or NF45_HV strains. Statistical analysis with Fisher test revealed a significant difference in 4 GO terms. DEGs in NF1_LV were mainly enriched in “GTP binding”, “GTPase activity” and “small GPTase binding” GO terms (all being mainly found to be downregulated). DEGs in NF45_HV were mainly enriched in “calcium ion binding” genes, being mainly downregulated (**Figure 4D**).

### 3.3 NF-brain infection altered the homeostasis in transcriptional regulation of the immune and neural system domains

Understanding how *N. fowleri* strains with different virulence traits impact the host response is crucial. Herein, we performed controlled infections with NF1_LV and NF45_HV strains and collected NF-infected mice brains at different days post-infection (**Figures 1**, **2**). Non-infected brains were used as controls.

#### 3.3.1 Transcriptome profiling of DEGs in NF-infected brains

The MA-plots presented in **Supplementary Figure S1** revealed that there was no normalization bias observed in the distribution of the genes according to fold-change and counts. From the PCA analyses (**Supplementary Figure S3**), we observed that the samples were clustered in 3 distinct groups (non-infected, infected with NF45_HV and infected with NF1_LV groups) and could be used for DEGs identification. For NF1_LV and NF45_HV-infected brain samples, we removed one biological replicate each as the reads were of low quality (data not shown). Heatmap illustrated that the (i) gene expression patterns for NF1-infected group were different from naive and NF45_LV-infected group but (ii) naïve and NF45_HV infected groups were only slightly different (**Supplementary Figure S3B**).

**Figure 5:**
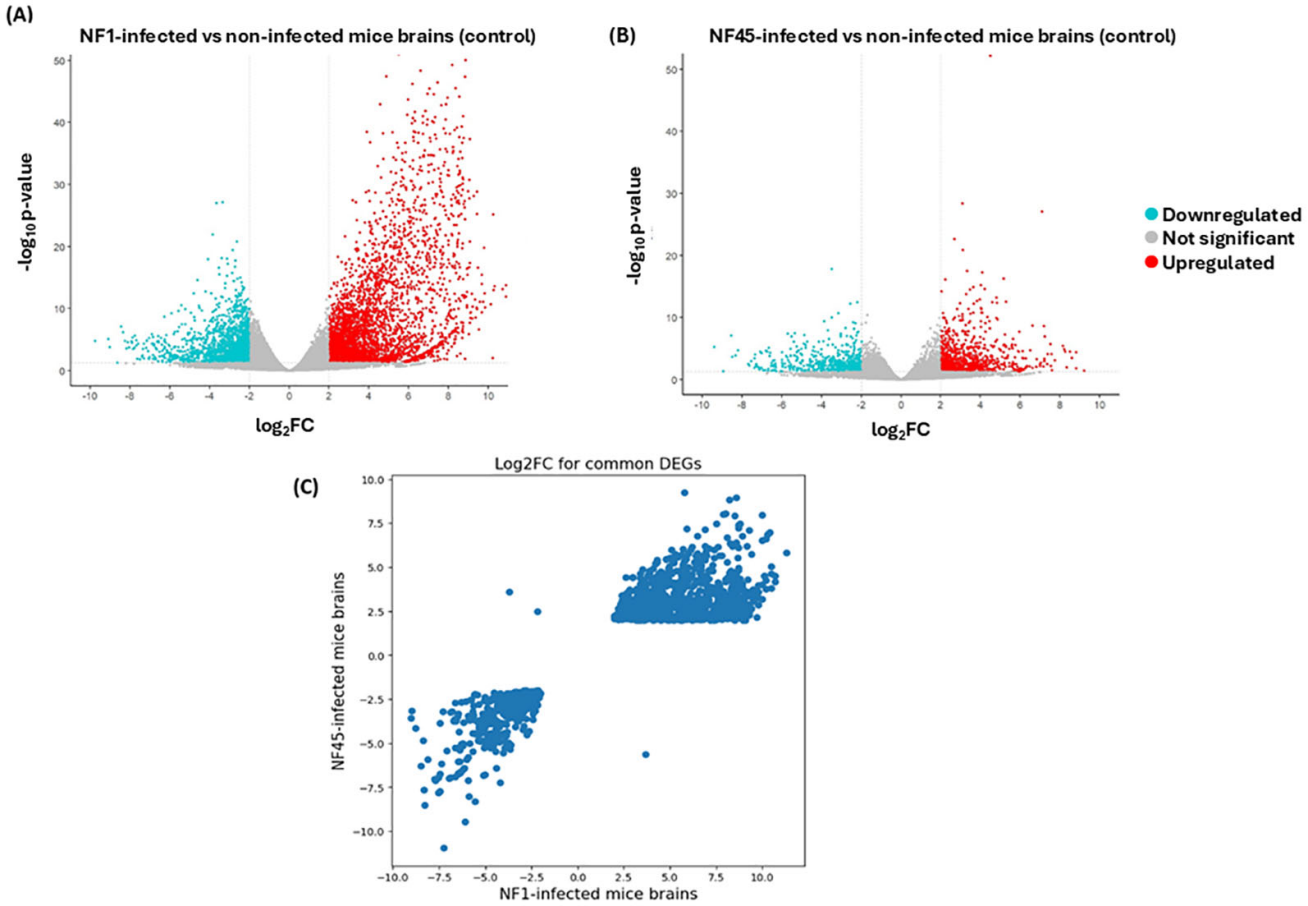
Differential Gene Expression analysis for NF1_LV and NF45_HV-infected brain samples compared to uninfected brains. **(A, B)** Volcano plots of DEGs. The up- and down-regulated genes are shown in color and the unchanged genes in grey. The dashed line indicates the threshold line for differential gene screening criteria and **(C)** log2FC values for common DEGs during NF1_LV and NF45_HV infected mice brains.

According to the criteria with p-value <0.05 and |log2FoldChange| ≥ 2, a total of 9149 genes were differentially expressed in mice infected with NF1_LV strain compared to the control, with 6372 and 2777 genes exhibiting up- and downregulated expression, respectively (**Supplementary Table S3**; **Figure 5A**). In NF45_HV- infected mice, 2743 DEGs were identified compared to the control, with 1744 and 999 being up- and downregulated respectively (**Supplementary Table S4**; **Figure 5B**). Globally, NF1_LV seemed to trigger a stronger reaction in the host (with higher number of DEGs) compared to NF45_HV (**Figure 5**). Additionally, we also observed that the mice infected with NF1_LV or NF45_HV shared 2018 DEGs. The expression of these common genes was found to be relatively similar independently of the NF strain (being up or down in both strains) but with expression levels (log2foldChange) was normally found to be higher during brain infection with NF1_LV (**Figure 5C**).

#### 3.3.2 Functional enrichment of DEGs

First, we identified the 5 top up and downregulated DEGs in NF1_LV and NF45_HV-infected mice brains. Our results (**Supplementary Table S3**) revealed that the top 5 upregulated genes in NF1_LV-infected brain were: CD antigen CD181 (neutrophil chemotaxis; immune response), Paired-Ig-like receptor A13 (cytokine-mediated signaling pathway), SLP adapter and CSK-interacting membrane protein (involved in major histocompatibility complex class II (MHC-II) signaling transduction), Cystatin A family member 2 (cell-cell adhesion) and Serine protease inhibitor A3M (Serpin A3M) (response to cytokine in eukaryotes) and Replication factor A protein 1 (major single-stranded DNA binding factor). The top 5 most downregulated mouse DEGs during NF1_LV infection were: Immunoglobulin kappa variable 8-28 (immune response), Prolactin receptor (PRL-R) (cytokine-mediated signaling pathway); LIM/homeobox protein Lhx9 (transcription factor), Transglutaminase-5 (peptide cross-linking) and Interleukin-22b (inflammatory response) (**Supplementary Table S3**).

The top 5 upregulated genes in NF45_HV-infected brain were: Fibrinogen alpha chain (immunity), Insulin gene enhancer protein ISL-1 (transcription), Interferon-activable protein 202 (immunity), Serine protease inhibitor A3M (response to cytokine) and Pleckstrin homology-like domain family A member 2 (mediator of apoptosis). The top 5 downregulated mouse DEGs during NF45_HV infection were: Immunoglobulin heavy variable V1-7 (innate immune response), Small integral membrane protein 22 (cytoskeleton organization), Transcription factor 21 (morphogenesis), Beta-defensin 11 (innate immune response) and Homeobox protein OTX2 (transcription factor) (**Supplementary Table S3**).

**Figure 6:**
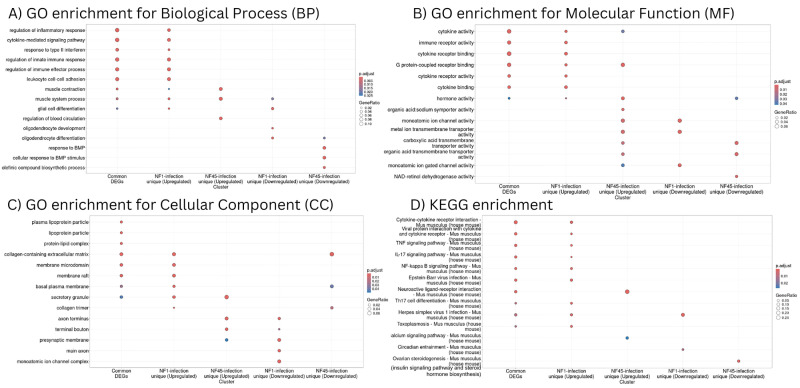
Functional enrichment analysis of differentially expressed genes in NF1_LV and NF45_HV-infected brain samples. **(A–C)** represent GO enrichment and **(D)** KEGG pathways of genes in 5 significant modules (common genes, up-regulated genes for NF1-infected brains, down-regulated genes for NF1-infected brains, up-regulated genes for NF45-infected brains and down-regulated genes for NF45-infected brains).

Next, the ORA analysis was performed with respect to Gene Ontology (Biological Process - **Figure 6A**; **Supplementary Table S5**, Molecular Function - **Figure 6B**; **Supplementary Table S6** and Cellular Component **-** **Figure 6C**; **Supplementary Table S7**) and KEGG pathways (**Figure 6D**; **Supplementary Table S8**). To perform this analysis, we considered only unique Uniprot identifiers. To compare further gene functional profiles during infection with NF1_LV and NF45_HV, 5 datasets were considered i) common DEGs detected both in NF1 or NF45-infected brains, ii) upregulated DEGs specific to NF1 infection, iii) downregulated DEGs specific to NF1 infection, iv) upregulated DEGs specific to NF45 infection and v) downregulated DEGs specific to NF45 infection. 53% of DEGs failed to map to NCBI identifiers.

Common DEGs detected during the brain infection by NF1_LV and NF45_HV and upregulated DEGs unique to NF1_LV infection had a similar functional profile (**Figures 6A–D**). GO and KEGG terms enriched were mainly related to immune response and muscle system, including cytokine signaling, binding, activity and cytokine-cytokine receptor interaction, regulation of inflammatory response, Tumor necrosis factor (TNF) and interleukin 17 (IL-17) signaling pathways. Upregulated genes in NF45_HV-infected brains included genes involved in muscle contraction and muscle system process, and regulation of blood circulation, with a strong impact in axon and presynaptic membrane, and related tendency towards transport and ion activity (namely calcium). Brain infection with NF1_LV resulted in the downregulation of genes encoding for muscle system process, glial cell and oligodendrocytes differentiation and oligodendrocytes and both strains did not show a clear functional specificity, with an impact in axons and ion transport. NF45_HV induced a strain-specific downregulation of genes related to response to BMP, collagen-containing extracellular matrix, and ion transport in infected brains.

#### 3.3.3 Construction of protein interaction networks and identification of “hubs” associated with NF infection

To further identify host proteins with functional relevance during NF infection, we analyzed protein-protein interactions between DEGs. Due to mice reaction specificity towards different NF strains, three scale-free networks were considered. Firstly, protein-protein interactions observed for DEGs which are common for both infections, were analyzed. This network represents a universal interactome response to NF infection regardless of the NF strain. It contains 799 nodes and 5635 edges and has an average clustering coefficient of 0.3. As typical for biological networks, most proteins in the network had only a couple of interactors, while a few proteins (“protein hubs”) have an outstanding number of them. In the universal response towards NF infection the most dominant proteins (hubs) were TNF-alpha, Interferon gamma (IFN-γ) and Interleukin 6 (IL-6). Their expression levels in NF1_LV-infected brain were significantly higher compared to NF45_HV infection, being some of the most upregulated genes in during NF1_LV infection (top 2%).

Afterwards, we created protein-protein interaction networks using strain-specific DEGs to provide key information on differences in host cell’s response towards amoeba infection (**Supplementary Figure S4**). Network created with DEGs present only in NF1_LV infected brains contained 2512 nodes and 29088 edges. The hubs associated to this PPI network included Actin (Actb), glycoprotein (CD4), Receptor-type tyrosine-protein (necessary for T-cells activation) and Interleukin-1β. The network built based on DEGs present only in NF45_HV infected brain revealed to be smaller and not connected, with 432 nodes and 547 edges and an average clustering coefficient of 0.156. The hubs included Paired box protein (Pax6, transcription factor), neuroendocrine (Gnas), and Insulin 2.

## 4 Discussion

**Figure 7:**
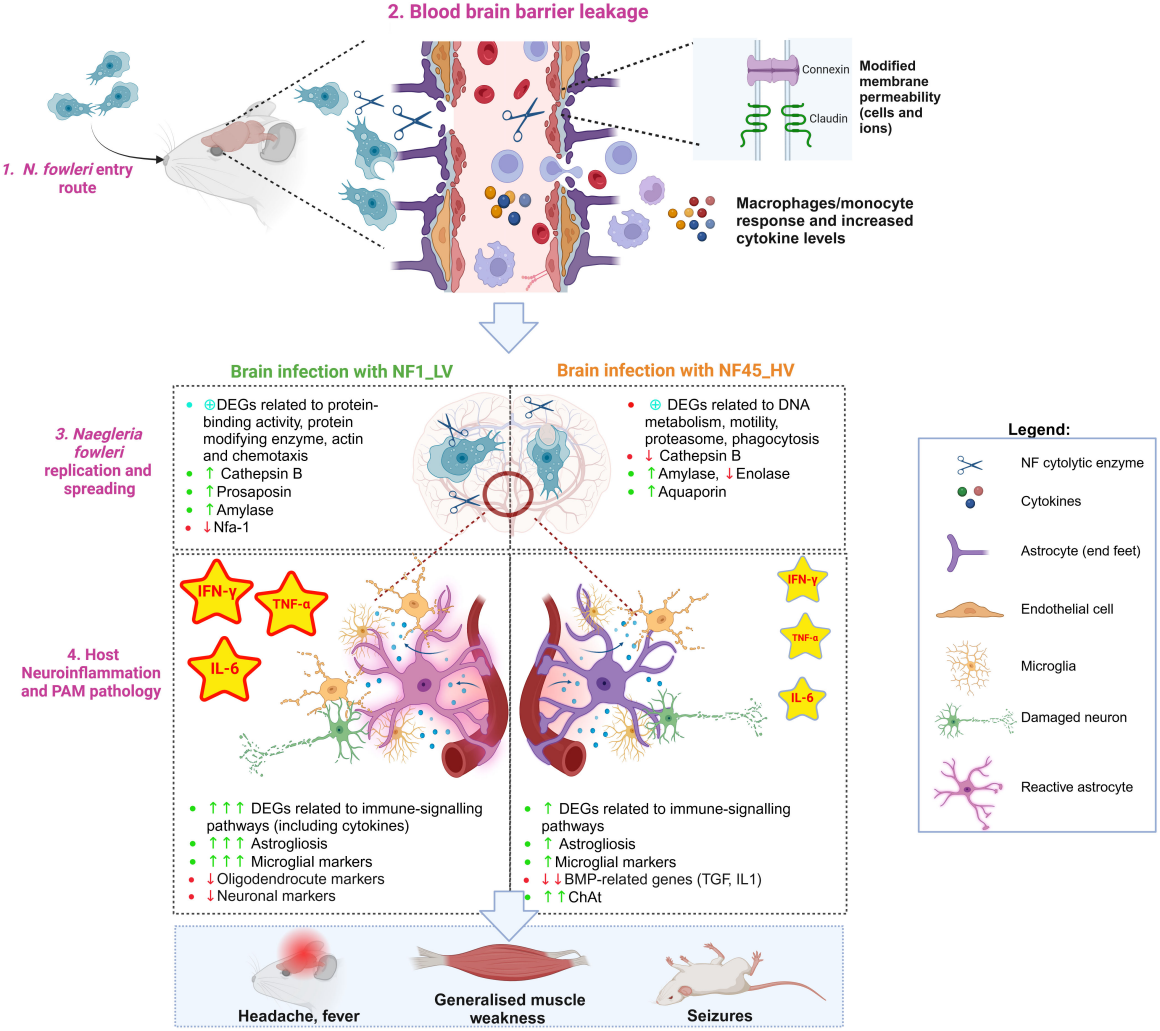
*Naegleria fowleri* infection process in mouse brain: the protozoa and the host perspectives (Created in BioRender. https://BioRender.com/p08c651).

Central nervous system (CNS) invasion is a devastating complication of a parasitic infection, and it involves the interplay of at least two components: (i) parasite properties (i.e., virulence and ability to establish productive replication in brain) and (ii) host defense responses and/or parasite ability to evade these responses. Despite several physical and immunological barriers that provide obstacles to such an invasion, some protozoa such as *N. fowleri* have developed the ability to surpass these barriers, leading to serious disease and often host death. To develop effective therapeutic approaches to treat PAM, it is crucial to identify the molecules and mechanisms underlying PAM disease. Herein, we used 2 newly isolated NF environmental strains of natural contrasting virulence phenotypes (NF1_LV and NF45_HV) to identify amoebae candidate virulence factors and study the host response while NF was replicating within the brain. Using comparative transcriptomic analysis, we found significant differences between the two NF strains, including distinct patterns within the mouse brain. Our findings summarized in **Figure 7** and described below, generate an important insight into specific genes and mechanisms underlying NF replication and spreading process inside the host and how the host responds to infection.

### 4.1 *Naegleria fowleri* entry route

*N. fowleri* trophozoites reaches the upper nasal mucosa using freshwater as a vehicle (**Figure 7**, Step 1). Once the amoeba is inside the nasal cavity, it can occasionally contact and cross the olfactory mucosa lining the upper regions of the nasal turbinates (Wellford and Moseman, 2024). After entering the olfactory mucosa, NF migrates along the olfactory nerves through the cribriform plate until it reaches the olfactory bulb within the brain. To penetrate the CNS, the amoeba must avoid local immune surveillance present along the olfactory route. Rojas-Hernández and colleagues observed a very early cellular exudate within the nasal turbinates hours after infection (Rojas-Hernández et al., 2004), however the patients affected by PAM do not show any apparent clinical signs and symptoms of nasal inflammation, and postmortem results do not reveal destruction of non-olfactory mucosa of the nasal cavity. This suggests that the phagocytic tissue damage by *N. fowleri* does not occur within the respiratory (non-olfactory) parts of the nasal cavity (Baig, 2015). It has also been shown in the mouse model that *N. fowleri* trophozoites can invade the olfactory neuroepithelium without causing cell death or alarming the immune system at 24h post-infection (Rojas-Hernández et al., 2004).

### 4.2 Disruption of the blood brain barrier integrity and function

When *N. fowleri* reaches the olfactory nerve bundles, the amoeba causes a series of damage in the host blood brain barrier (BBB) (**Figure 7**, Step 2).

#### 4.2.1 Disruption of BBB physical integrity and function

*In vitro* experiments using rat brain microvascular endothelial cells as a model from BBB revealed that NF disrupts the tight junction proteins (in particular claudin) (Coronado-Velázquez et al., 2018). Our *in vivo* study showed that NF infection triggers molecular responses in different cell types with critical roles in BBB physical integrity and function, namely astrocytes and endothelial cells (**Figure 7**, Step 2). NF infection resulted in a significant decrease in the expression of genes encoding cell adhesion molecules such as Cldn-related proteins 10 and 15, which are important in sealing tight junctions at the brain barrier. We also observed a differential expression of genes encoding for Connexin, Cadherin and Desmocollin, conferring barrier restrictions for permeability between endothelial and astrocyte base feet. Interestingly, the increased expression of genes encoding for Connexin could be related to hemichannel opening and the activation of intracellular calcium concentration dynamics, contributing to BBB physical leakage (De Bock et al., 2022).

#### 4.2.2 Endothelial cells and astrocytes reaction

During infectious processes, inflamed endothelial cells and reactive astrocytes are known to upregulate the expression of adhesion molecules that facilitate the migration of circulating peripheral immune cells (monocytes/macrophages and lymphocytes) and neuroimmune-related substances across the BBB (Yu et al., 2022). Previous *in vitro* studies revealed that NF-induced BBB leakage induced the expression of adhesion molecules and inflammatory mediators such as VCAM-1 and ICAM-1 (Coronado-Velázquez et al., 2018). Our transcriptomic data demonstrated increased levels of *Vcam1* (only for NF1_LV) and *Icam-1* (in both cases), but also the activation of other endothelial markers such as *B2m*, *H2-D1*, *H2-K1*, Toll-like receptor 4 and CD14. Endothelial cells can also constitutively secrete IL-6, prostaglandin, and nitric oxide during infectious processes (Yu et al., 2022). We found elevation of several DEGs encoding for nitric oxide synthase (NOS), prostaglandin E2 receptor, IL-6 (being a protein “hub”) and its IL-6 receptors, particularly in NF1_LV-infected brain. Increased levels of IL-6R present in astrocytic end feet could lead to reactive astrocytic state (De Bock et al., 2022), as indicated by the expression of astrogliosis-associated DEGs such as *Gfap, Slc6a11*, and *Kcnn4* (for both NF1_LV and NF45_HV infections), *Ntsr2, Ntm* and *Aldoc* (only detected during NF1_LV), and *Slc1a3 and Fam107a* (only in NF45_HV). These reactive astrocytes can upregulate proinflammatory and cytotoxic pathways, and consequently, produce a range of substances associated with barrier leakage (De Bock et al., 2022) (**Figure 7**, Step 2). Our dataset also indicated substantial downregulation of bone morphogenetic proteins (BMPs)-related genes during NF45_HV. BMPs which include the cytokines TGF-β and IL-1 are known to induce nitric oxide (NO) production by astrocytes and BMPs further promote the inflammatory phenotype of endothelial cells. Downregulation of these genes could allow NF to control inflammation at later stages of the infection. Interestingly, we also detected the expression of genes encoding for Serum amyloid A (SAA) in the mouse brain. Recent work has shown that SAA proteins can enter the brain by crossing the intact BBB, impairing its function, namely by inducing the expression of cytokines and promoting astrogliosis (Erickson and Mahankali, 2024). This overall dysfunction at the BBB level would lead to neuroinflammation and neurodegeneration, as further discussed in Section 4.4.

### 4.3 Active replication and spreading of *N. fowleri* in the brain

Alongside with the BBB leakage, NF begins to invade the inferior surface of the frontal lobe of the brain. Our results in mice showed that NF45_HV has spread within the entire brain cerebrum at 4 days post-infection, while NF1_LV is still mainly located at the olfactory bulb, which suggested a slower rate for invasion. To spread within the brain, NF trophozoites can use different strategies involving contact-dependent and/or and contact-independent mechanisms (**Figure 7**, Step 3).

#### 4.3.1 Phagocytosis (contact dependent)

Direct damage to host cells by the phagocytic activity of *N. fowleri* has been recognized as a major pathogenic mechanism (Cursons and Brown, 1978; Marciano-Cabral and Cabral, 2007). While the current understanding of events of the phagocytic pathway in NF are very limited, two proteins Nfa1 (also called Hemerythrin-like protein) and actin are known to play a critical role in food cup formation and phagocytosis (Shin et al., 2001; Kang et al., 2005; Sohn et al., 2010; Velle and Fritz-Laylin, 2020). Herein, the *nfa1* gene was found to be downregulated by NF1_LV; previous reports have shown that the blocking Nfa1 (using a specific antibody) caused a decrease in the cytotoxicity of *N. fowleri* against target cells (Jung et al., 2008). Phagocytosis requires a tight regulation of the cell cytoskeletal network dynamics, and for this, several proteins are required namely Cofilin, Clathrin, Filamin, Spectrin, Vimentin, Profilin, Arp2/3 complex and Rho family GTPases (Kamil et al., 2022). Our transcriptomics data revealed that NF45_HV overexpressed the genes encoding for Severin, Filamin, Clathrin and downregulated the expression of Profilin, formin-like protein and Myosin I. Our *in vivo* results are partly in agreement with the previous observations by Zysset-Burri and colleagues, as they observed that actin-related protein such as Villin, Severin, Myosin and Formin were more abundant *in vitro* cultured highly virulent *N. fowleri* (Zysset-Burri et al., 2014). Calcium binding proteins are also required to regulate the progression of phagocytosis (Nunes and Demaurex, 2010; Babuta et al., 2020). For instance, the Calmodulin-like calcium binding protein EhCaBP3 of *Entamoeba histolytica* has been shown to be directly involved in disease pathomechanism (Aslam et al., 2012). The use of loperamide (a calmodulin inhibitor) was seen to prevent the damage to the human cells HBMEC by *N. fowleri* trophozoites even after passage of 12 h, hampering the activation of the host immune response (Baig, 2015). Our transcriptomics data revealed that NF45_HV strain has more calcium ion binding (namely calmodulins) which are downregulated, probably because the infection is at a highly advanced state (as observed in **Figure 2D**). These results suggest that NF45_HV and NF1_LV possess different “rates” for phagocytosis in the brain, possibly resulting in differential host cell damage and concomitant stimulation of the immune system.

#### 4.3.2 Release of cytolytic molecules (contact independent)

*N. fowleri* can induce host cell and nervous system destruction upon the release of cytolytic molecules, including pore-forming protein, acid hydrolases, phospholipases, neuraminidases, phospholipolytic enzymes and cysteine proteases (Aldape et al., 1994; Herbst et al., 2002, 2004; Visvesvara et al., 2007; De Jonckheere, 2011; Zyserman et al., 2018). Our transcriptomic dataset showed that genes involved in protein-binding activity modulation and protein modifying enzymes are more abundant in NF1_LV (which agrees with a higher number of DEGs involved in host immune response, as discussed in Sections 4.2 and 4.4). More specifically, Prosaposin (also termed Naegleriapore A) and Cathepsin-like proteases were found to be upregulated in NF1_LV gene data set. In NF45_HV-infected brains, Cathepsin B proteases were found to be downregulated which could be related to the fact that NF45_HV does not require more “pre-digestion” of the host cells when it is already widespread in the brain. While other cathepsin-like proteins (namely Cathepsin A or Nf314) were found to be upregulated in mouse-passaged *N. fowleri* (Hu et al., 1992), we did not detect the differential expression of these genes in both NF1_LV and NF45_HV.

#### 4.3.3 Cytoskeleton-related DEGs

As amoebal infection progresses within the brain, it is expected that NF genes involved in motility/chemotaxis, cell division process, oxidative stress, protein synthesis/recycling/modification and metabolism are differentially modulated. *N. fowleri*’s pathogenesis involves actin-mediated cell motility (Velle and Fritz-Laylin, 2020; Fulton, 2022; Velle et al., 2022). While we detected 20 actin-related DEGs in NF1_LV and 9 in NF45_HV (some of them being also related to the phagocytic process described above), the cytomotive filament myosin was found to be downregulated in both NF1_LV and NF45_HV. Interestingly, our results also revealed the presence of 6 DEGs encoding for cilia and flagella-like organelles in NF45_HV (all being downregulated) while 6 DEGs were overexpressed in NF1_LV. While it is unlikely that *N. fowleri* would “swim” in the brain using flagella, these genes could be related to signal transduction, allowing to sense its environment. It has been suggested that NF can also selectively ‘sense’ neurotropic factors (Jamerson et al., 2012; Baig, 2016). In our data set, we detected several DEGs involved in chemotaxis, in particular in NF1_LV, promoting the migration of NF1_LV towards the brain.

#### 4.3.4 DNA metabolism related DEGs

Current knowledge on *Naegleria* cell cycle progression and control is scarce. Our transcriptomics data set revealed the differential expression of several genes related to cell cycle progression and control, namely DNA Damage Responses, DNA replication, mitotic spindle checkpoint. Of particular interest are E3 ubiquitin ligase (with a crucial role in protein ubiquitination, as discussed below) and Serine/threonine NEK kinases which could an impact the amoeba life cycle progression and survival, as previously observed for malaria parasite (Singh et al., 2023) and *Giardia* (Hennessey et al., 2020). Herein, we detected elevation of DEGs encoding for NEK protein in NF1_HV which probably is in agreement that this strain is actively replicating in the brain while NF45_HV has reduced its replication rate.

#### 4.3.5 Antioxidant and stress response systems

*N. fowleri* must possess an efficient antioxidant system to survive the invasion of oxygenated brain tissues and survive to the aerobic stress caused by the host immune response. Our transcriptomics results indicated that both NF1_LV and NF45_HV actively upregulated thioredoxin-related genes while inside the brain, while DEGs encoding for ruberythrin and hemerythrin (Nfa1) [potentially involved in oxygen sensing (Dereeper et al., 2023)] were found to be downregulated in NF1_LV. It is possible that *N. fowleri* used alternative strategies such as protein posttranslational modifications (PTMs) to handle stress responses. Herein, we found a strong modulation of DEGs encoding for E3 ubiquitin-protein ligase in both strains (related to ubiquitination) and proteasome. In other protozoa such as *Giardia lamblia, Leishmania* spp.*, Trypanosoma* spp.*, Toxoplasma gondii, Plasmodium* spp.*, Entamoeba* spp. and in the free-living amoebae*, Acanthamoeba castellanii and Dictyostelium discoideum*, the role of the 26S and 20S proteasome has been demonstrated in cellular processes such as proliferation, differentiation, virulence and in the stress response [reviewed by (Guzmán-Téllez et al., 2020)]. Previous work has revealed that inhibition of the proteasome can also affect the proliferation of *Naegleria* sp trophozoites (Guzmán-Téllez et al., 2020). Herein, we detected 17 DEGs related to the proteasome system (namely 26S and 20S subunits), 16 being up-regulated in NF45_HV and one being down-regulated in NF1_LV. This high number of proteasome related genes in NF45_HV suggest the proteasome could be an interesting target, as proposed for other protozoa.

#### 4.3.6 Amoeba metabolism during infection

The metabolic needs of *N. fowleri* during human infection remain unresolved. Experiments using non-pathogenic *N. gruberi* trophozoites revealed that the parasite would prefer to oxidize fatty acids to generate acetyl-CoA, rather than use glucose and amino acids as growth substrates (Bexkens et al., 2018). Recently, several genes involved in metabolism of both lipids and carbohydrates were shown to be upregulated in mouse-passaged *N. fowleri*, being possibly related to the amoeba pathogenesis (Herman et al., 2021). However, recent studies suggest that a Enolase, a key enzyme glycolysis and gluconeogenesis, would be essential in *N. fowleri*, as Enolase inhibitors were shown to be lethal for the amoeba (Milanes et al., 2024). Our results revealed that a Enolase family member was found to be downregulated in NF45_HV, which suggests that the amoeba has a reduced glucose metabolism at an advanced state of brain infection and/or it can use an alternative process to obtain energy from carbohydrates. Other enzymes involved in carbon metabolism such beta and alpha amylase have shown to be important in protists, namely in *Entamoeba histolytica*. Indeed, when glucose levels in the colonic lumen are low, virulent *E. histolytica* utilize glycoside hydrolase (β-amylase, which is absent from humans) to use host mucus glycans as carbon source (Thibeaux et al., 2013). Herein, we detected both alpha and beta-amylases, both being up-regulated in NF1_LV. As these enzymes do not exist in the human host, they are interesting candidates for drug development against NF. We also detected the presence of O-methyl transferases (2 isoforms, up-regulated in NF45_HV), which could increase the diversity of natural products made.

### 4.4 Host neuroinflammation and PAM pathology

#### 4.4.1 Immune reaction from the host

Neutrophils have been considered as the primary mediators of the rapid innate host defense against *N. fowleri* (Ferrante et al., 1988). Herein, we detected increased expression of marker genes such as *Cd44*, *Ccl24*, *Ccl7*, *Cd74*, *S100a*-related genes, *Wnt10b* in both infection by NF1_LV and NF45_HV and *Cd36, Cd47, Mrc1*, in particular for NF1_LV, indicating the presence of infiltrating macro/monophages during the infectious process (**Figure 7**, Step 2). Our transcriptomics results also revealed the modulation of DEGs related to several innate immune-related pathways (in particular for NF1_LV), including the RIG-I-like receptor signaling pathway (*Irf7*, TRIM25 and the pattern recognition receptors DHX58 and IFIH1), interferon (IFN) alpha and beta pathways, the Toll-like receptor signaling pathway (with 8 different TLR being overexpressed in NF1), the JAK-STAT signaling pathway, NLRP3 inflammasome and the expression of the IFN-induced transmembrane proteins (IFITMs). We observed that *Ifitm1*, *Ifitm2*, *Ifitm3*, *Ifitm5* and *Ifitm6* were highly upregulated during NF1_LV infection while only *Ifitm1*, *Ifitm3*, *Ifitm6* were upregulated during NF45_HV exposure and at a lower level. This indicates that IFN signaling and inflammation may not be homogeneous during early and late states of infection, and have a NF-specific effect. We also observed the upregulation of DEGs related to antigen presentation such as *Tap1* and *Tap2*, in particular during infection with NF1_LV, suggesting the activation of the adaptive immune system. Indeed, our results showed that NF1_LV infection resulted in increased expression of markers genes (namely *Cd4*) encoding for lymphocytes (T and B cells).

#### 4.4.2 Astrocyte and oligodendrocytes reaction

Astrocytes can also provide, along with oligodendrocytes, nutritional support for neurons (Zang et al., 2022). Our results showed that DEGs encoding for oligodendrocytes markers genes such as *Mbp*, *Mog* and *Plp1* are strongly downregulated during NF1_LV infection, suggesting oligodendrocyte damage. This could be due to the NOS gene activation (either by endothelial cells or astrocytes), resulting from IFN-γ, TNF-α and IL-1β active secretion (**Figure 7**, Step 4).

#### 4.4.3 Microglia importance

In response to stress, oligodendrocytes are known to induce the expression of chemoattractants to actively recruit microglia to damaged tissues. Herein, we detected increased levels of *Cxcl10*, *Ccl2* and *Ccl3* during NF1_LV and NF45_HV infection and a strong activation of microglia in particular during NF1_LV infection, with the upregulation of microglial markers (such as *P2ry6, Selplg and Tmem119*) and disease-associated microglia (DAM) genes (namely *Tyrobp*, *Trem2* and *Ctss).* According to our dataset, the activation of microglia would result in the secretion of excessive amounts of pro-inflammatory cytokines and neurotoxic molecules, such as IL-1, IL-6, and TNF-α which in turn contribute to the degeneration and death of neuronal cells (as indicated by the decrease of neuronal markers such as *Syt 1*, *Syt2* and *Syt7* and *Calb*), (**Figure 7**, Step 4). While the role of microglia in PAM disease has been partly studied *in vitro* (Oh et al., 2005; Marciano-Cabral and Cabral, 2007; Lee et al., 2011; Retana Moreira et al., 2024), our results clearly showed the *in vivo* importance of microglia in PAM disease.

#### 4.4.4 Possible origin of PAM symptoms

As NF infection progressed, the continuous stimulation with high cytokine concentrations (in particular IL-6, IFN-γ and TNF-α) led to the transformation of microglia/astrocytes/endothelial cells into a dysfunctional state, generating a generalized inflammation and the loss of neural support functions and resulting in PAM symptoms. PAM symptom such as neck stiffness could be attributable to the inflammation [in particular due to IL-6 (Yang et al., 2024)], as the swelling around the spinal cord makes it impossible to flex the muscles. On the other hand, the loss of brain tissue (BBB, astrocytes, neurons, microglia, etc.) could strongly contribute to the appearance of symptoms such headache, nausea, photophobia, seizures, vomiting and cognitive impairment. In NF1_LV-infected brains, where the infection is still at “early stage”, we observed in a strong downregulation of many nervous system-related genes such as *Adam22* (related to adult locomotory behavior), *Cacnb4* (linked to adult walking behavior and detection of light stimulus involved in visual perception), *Zic1* (related to walking behavior and inner ear morphogenesis), and *Gabra1* (mutation of these gene is associated with neurodevelopmental defects and epilepsy) possibly linked to the above mentioned symptoms. Interestingly, during NF45-infection, we notice the upregulation of DEG encoding for spinal motor neuron-specific marker choline acetyltransferase (ChAT), the enzyme responsible for the biosynthesis of neurotransmitter acetylcholine. Excessive accumulation of acetylcholine (ACh) at the neuromuscular junctions and synapses would cause PAM symptoms such as cramps, muscular weakness, and blurry vision (**Figure 7**, Step 4).

## 5 Conclusion

Our comparative transcriptomic analysis of NF strains with different virulence paired with phenotypic data on their *in vivo* pathogenesis uncovers new angles to understand the pathobiology of a rare but highly fatal protozoa and PAM disease. This comparative analysis of two distinct NF strains with different rate of progression within the mouse brain provided key information about early and late infection time points that integrate the interaction between host and parasite. While Baig (2015) suggested that brain infection with *N. fowleri* results in an extensive brain damage largely caused by the host immune response rather than the amoeba, our results showed that the both the parasite and the host played key roles in PAM disease. NF strains showed to differently express genes which are crucial for their replication and spreading within the brain, some of them being potential drug targets such as amylase, and calmodulin, proteasome and NEK proteins. We demonstrated that NF infection triggered transcriptional responses linked to physical damage at the brain blood barrier and changes in brain-infiltrating/resident macrophages/monocytes and lymphocytes leading to an exacerbated modulation of genes (in particular during NF1-infection) mainly linked to the host active immune response, inflammation and neurodegeneration. From the above, it is clear that a dual approach for PAM infection treatment should be used by reducing NF progression and limiting the neuronal and other organ damages that occur by selectively containing the immune system (by developing newer immuno-modulatory therapies namely targeting IL-6, for instance) that would lead to a greater margin of safety and clinical efficacy and increase the chances of winning the fight against this rare but fatal disease.

## Data Availability

The data presented in the study were deposited in the NCBI database under BioProject accession number PRJNA1181852. The raw RNA-seq reads and genome data can be accessed in the Sequence Read Archive (SRA) associated with this BioProject. *Naegleria fowleri* ITS and 18S sequences for NF1 and NF45 strains have also been deposited on NCBI and are available in GenBank under accession numbers: PQ573549, PQ573550, PQ571242 and PQ571243.

## Supplementary material

Supplementary Table 1 ITS and 18S sequences of NF1 and NF45 strains isolated in natural waters in Guadeloupe.

Supplementary Table 2 List of the DEGs in NF1_LV and NF45_HV strains (after vs before brain infection) (the complete list will be provided after manuscript acceptance).

Supplementary Table 3 DEGs between NF1_LV-infected and non-infected mouse brains.

Supplementary Table 4 DEGs between NF45_LV-infected and non-infected mouse brains.

Supplementary Table 5 GO enrichment for Biological process of DEGs in mice infected with NF1_LV or NF45_HV.

Supplementary Table 6 GO enrichment for Molecular function of DEGs in mice infected with NF1_LV or NF45_HV.

Supplementary Table 7 GO enrichment for Cellular component of DEGs in mice infected by NF1_LV or NF45_HV.

Supplementary Table 8 KEGG enrichment for DEGs in mice infected by NF1_LV or NF45_HV.

Supplementary Figure 1 MA plots of differential gene expression levels in RNA-seq data for NF and mice brain samples. The MA plot shows the distribution of the genes according to fold-change (y axis) and counts (x axis). Each dot represents one gene, and the blue color indicates the significant differentially expressed genes (as classified by DESeq2) between two conditions for the following set of samples: NF1_LV **(A)**, NF45_HV **(B)**, Mouse brains infected by NF1_LV **(C)** and NF45_HV **(D)**.

Supplementary Figure 2 Principal component analysis (PCA) plots and sample-to-sample distance heatmap of gene expression levels in RNA-seq data for NF samples. **(A)** PCA for NF1_LV before and after infection showing two distinct group **(B)** PCA for NF45_HV before and after infection showing two distinct group **(C)** Sample distance Heatmap the color of the heatmap indicates the correlation of gene expression levels in the sample, lighter blue represents low correlation, and darker blue represents high correlation.

Supplementary Figure 3 Principal component analysis (PCA) clustering diagram **(A)** and correlation heatmap of gene expression levels **(B)** in RNA-seq data for NF1_LV and NF45_HV brain infected samples. The x-label represents the variance contribution rate of principal component 1, and the y-label represents the variance contribution rate of principal component 2. The color of the heatmap indicates the correlation of gene expression levels in the sample, light blue represents low correlation, and dark blue represents high correlation. R1, R2 and R3 indicates the different biological replicates.

Supplementary Figure 4 Degree distribution on the log-log scale for protein-protein interactions networks built on 1) DEGs common for both strains B) DEGs specific for NF1_LV infection, C) B) DEGs specific for NF45_LV infection.
