# Supplementary material for: Transcriptomic profiling of “brain-eating amoeba” Naegleria fowleri infection in mice: the host and the protozoa perspectives
Source: Front Cell Infect Microbiol. 2024 Dec 16;14:1490280. doi: 10.3389/fcimb.2024.1490280 (PMC11682717; doi:10.3389/fcimb.2024.1490280)
Supplement: Supplementary Table 1 — ITS and 18S sequences of NF1 and NF45 strains isolated in natural waters in Guadeloupe. [file Table1.pdf]

Table S1. ITS and 18S sequences of *Naegleria fowleri strains 1 and 45* isolated in Guadeloupe

Accession number	Sample name	Sequence t	Sequence	Description	Max Score	Total Score	Query Cove	E value	Per. Ident	Accession	Genotype
PQ573549	NF_NF1_2020 (Morphy, GP)	ITS1	GTAAAAAAGGTGTATGGTAAAAAAGGTGAAAACCTTTTTTCCATTTA<br>CAAAAAATAACTCTGTGCAATGGAGCACACGGCTCGTGTATCGAT<br>GAAGCCCGCGGCAAAAAGCGATATGTAATGAGATTCGTTAGCCTC<br>GAGATTCATCAAATTGGTGAACACAGTCTGGACCTCGCAAGAGGT<br>ACTTACGTTAGAGTGCTAGTTTTATATCAATTGATACTGGTAAAAG<br>GTGTATTTAATCAATAGATTTTTACGCCCTAGCTGGTTATGCCGGA<br>TTCTCTTTGAGAAAACCGGATTGTCCCATTTGAAATTTTTTCAAATG<br>GTCAATCTTTTATTTAACTAGCCTCTAATGTGAGAGGGTACCCCCT<br>GGATTTAAGCATATTAATAAGGGGAGGAAAAA	Naegleria fowleri isolate RA internal transcribed spacer region, partial sequence	737	737	100%	0.0	100.00%	ON197492.1	2
PQ571242		18S	TGATTGACAGGTTAATAGCCCTTTCTTGATTGTGTGGTGGGTAGTG<br>CATGGCCGTTTCCAGTTCGTGGAGTGATCTGTCTTGTTAATTCAGA<br>TAACGAACGAGACCTAAGCCTTTAACTAGCCGTAGGCCTCTTCCTT<br>CGGGGAGGGGTTAGTTTGTCGGGACTGGCTTTTAGCCTGTTCCAA<br>AACCTACGTGACTTTTGTCAGCTTCTTAAAGGGACTTCATTCGTTC<br>TTGTAGAATGAGGAAGATTTAGGCCATAACAGGTCTGTGATGCAA	Naegleria fowleri 18S ribosomal RNA gene, partial sequence	503	503	99%	1,00E-137	100.00%	KY062165.1	-
PQ573550	NF_NF45_2020 Grosse Corde, GP)	ITS1	ATGGTAAAAAGGTGTATGGTAAAAAAGGTGAAAACCTTTTTTCCAT<br>TTACAAAAAAATAACTCTGTGCAATGGAGCACACGGCTCGTGTATCG<br>ATGAAGCCCGCGGCAAAAAGCGATATGTAATGAGATTCGTTAGCC<br>TCGAGATTCATCAAATTGGTGAACACAGTCTGGACCTCGCAAGAG<br>GTACTTACGTTAGAGTGCTAGTTTTATATCAATTGATACTGGTAAAA<br>GGTGTATTTAATCAATAGATTTTTACGCCCTAGCTGGTTATGCCGG<br>ATTCTCTTTGAGAAAACCGGATTGTCCCATTTGAAATTTTTTCAAAT<br>GGTCAATCTTTTATTTAACTAGCCTCTAATGTGAGAGGGTACCCCC<br>TGGATTTAAGCATATTAATAAGGGGGAGGAAAAAA	Naegleria fowleri isolate RA internal transcribed spacer region, partial sequence	732	732	99%	0.0	99.50%	ON197492.1	2
PQ571243		18S	GTTTGATTGACAGGTTAATAGCCCTTTCTTGATTGTGTGGTGGGTA<br>GTGCATGGCCGTTTCCAGTTCGTGGAGTGATCTGTCTTGTTAATTC<br>AGATAACGAACGAGACCTAAGCCTTTAACTAGCCGTAGGCCTCTTC<br>CTTCGGGGAGGGGTTAGTTTGTCGGGACTGGCTTTTAGCCTGTTC<br>CAAAACCTACGTGACTTTTGTCAGCTTCTTAAAGGGACTTCATTCG<br>TTCTTGTAGAATGAGGAAGATTTAGGCCATAACAGGTCTGTGATGC<br>A	Naegleria fowleri 18S ribosomal RNA gene, partial sequence	508	508	99%	2,00E-139	100.00%	KY062165.1	-
