# Supplementary figures and images for: Transcriptomic profiling of “brain-eating amoeba” Naegleria fowleri infection in mice: the host and the protozoa perspectives
Source: Front Cell Infect Microbiol. 2024 Dec 16;14:1490280. doi: 10.3389/fcimb.2024.1490280 (PMC11682717; doi:10.3389/fcimb.2024.1490280)

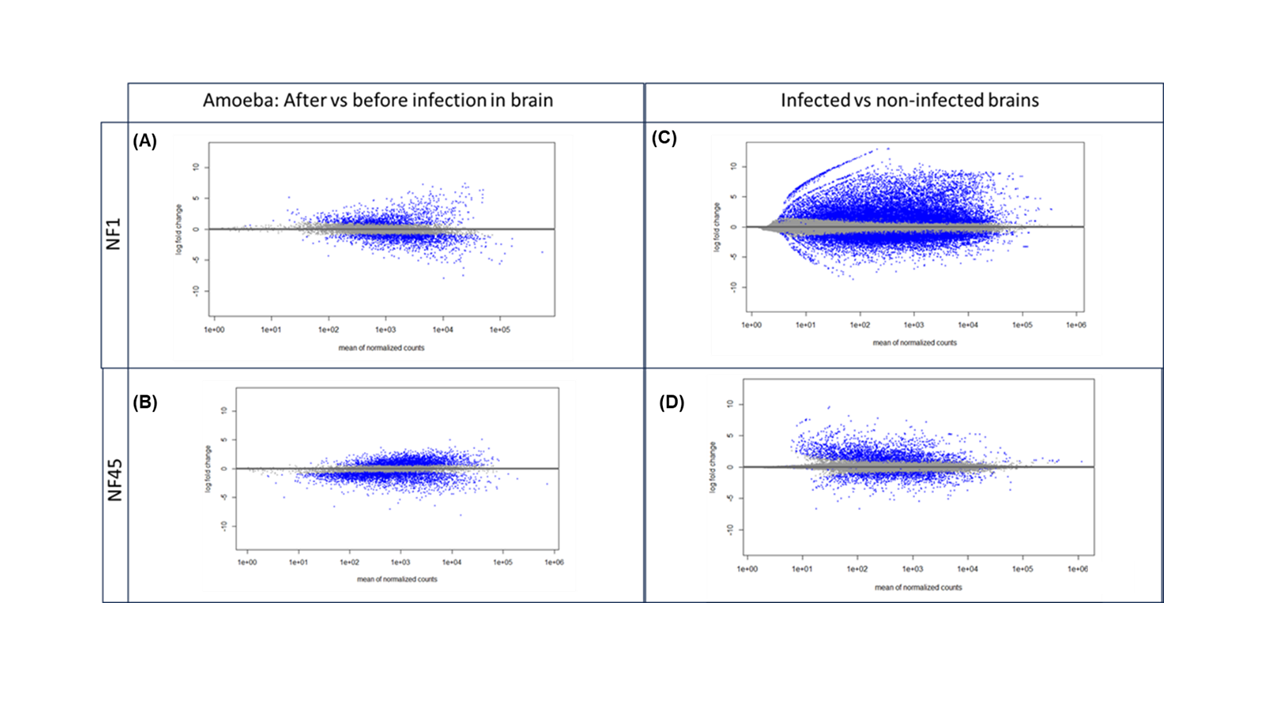

Supplement: Supplementary Figure 1 — MA plots of differential gene expression levels in RNA-seq data for NF and mice brain samples. The MA plot shows the distribution of the genes according to fold-change (y axis) and counts (x axis). Each dot represents one gene, and the blue color indicates the significant differentially expressed genes (as classified by DESeq2) between two conditions for the following set of samples: NF1_LV (A), NF45_HV (B), Mouse brains infected by NF1_LV (C) and NF45_HV (D). [file Image1.png]

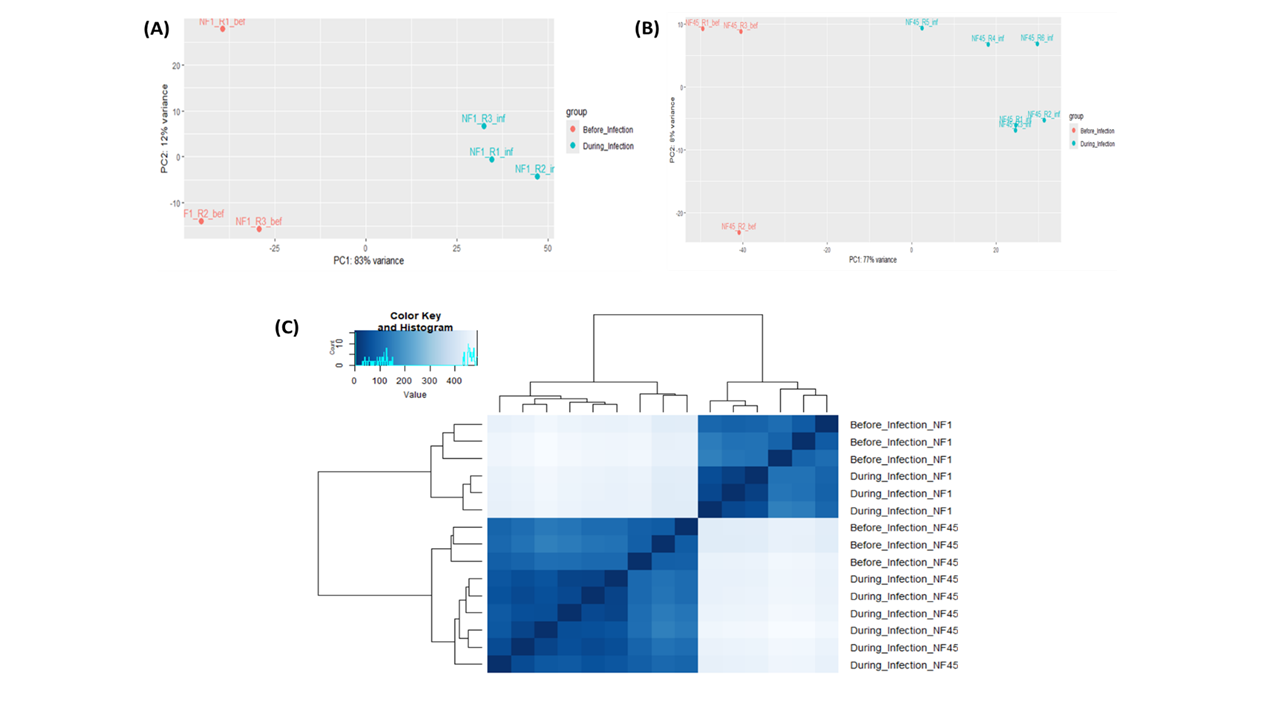

Supplement: Supplementary Figure 2 — Principal component analysis (PCA) plots and sample-to-sample distance heatmap of gene expression levels in RNA-seq data for NF samples. (A) PCA for NF1_LV before and after infection showing two distinct group (B) PCA for NF45_HV before and after infection showing two distinct group (C) Sample distance Heatmap the color of the heatmap indicates the correlation of gene expression levels in the sample, lighter blue represents low correlation, and darker blue represents high correlation. [file Image2.png]

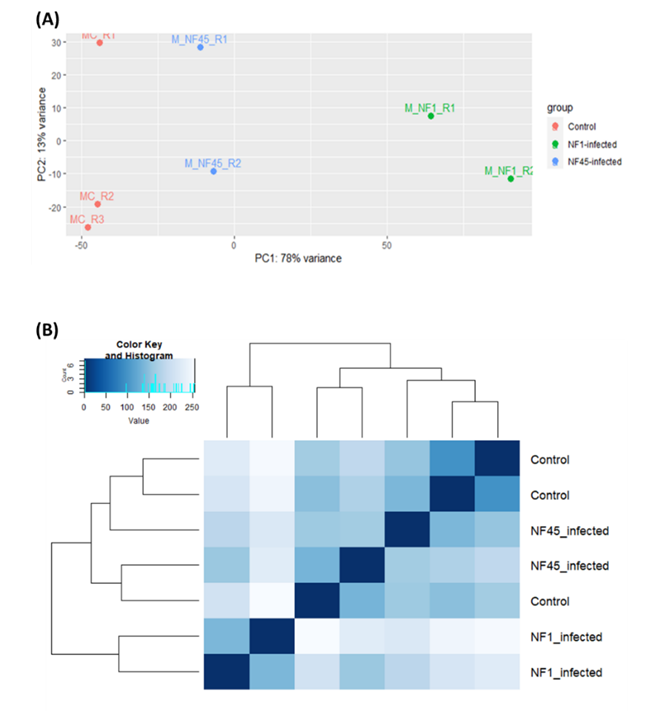

Supplement: Supplementary Figure 3 — Principal component analysis (PCA) clustering diagram (A) and correlation heatmap of gene expression levels (B) in RNA-seq data for NF1_LV and NF45_HV brain infected samples. The x-label represents the variance contribution rate of principal component 1, and the y-label represents the variance contribution rate of principal component 2. The color of the heatmap indicates the correlation of gene expression levels in the sample, light blue represents low correlation, and dark blue represents high correlation. R1, R2 and R3 indicates the different biological replicates. [file Image3.png]

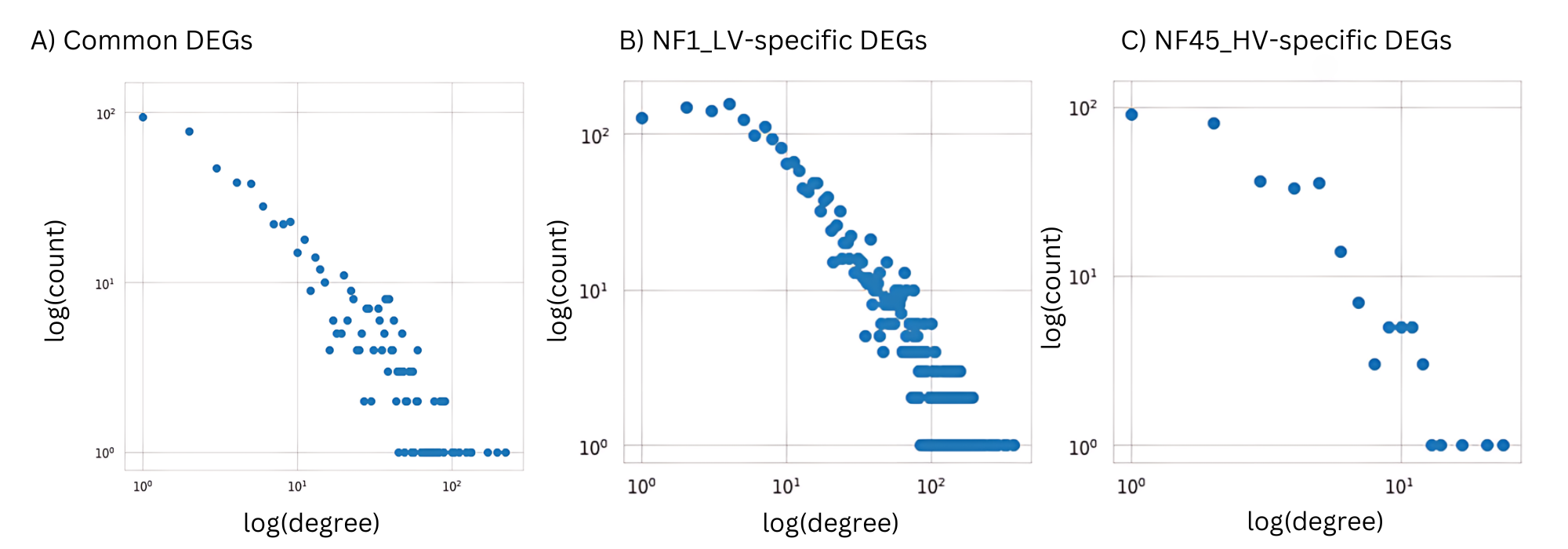

Supplement: Supplementary Figure 4 — Degree distribution on the log-log scale for protein-protein interactions networks built on 1) DEGs common for both strains B) DEGs specific for NF1_LV infection, C) B) DEGs specific for NF45_LV infection. [file Image4.png]
